# Permanent draft genome sequence of *Dethiosulfovibrio peptidovorans* type strain (SEBR 4207^T^)

**DOI:** 10.4056/sigs.1092865

**Published:** 2010-08-20

**Authors:** Kurt LaButti, Shanmugam Mayilraj, Alicia Clum, Susan Lucas, Tijana Glavina Del Rio, Matt Nolan, Hope Tice, Jan-Fang Cheng, Sam Pitluck, Konstantinos Liolios, Natalia Ivanova, Konstantinos Mavromatis, Natalia Mikhailova, Amrita Pati, Lynne Goodwin, Amy Chen, Krishna Palaniappan, Miriam Land, Loren Hauser, Yun-Juan Chang, Cynthia D. Jeffries, Manfred Rohde, Stefan Spring, Markus Göker, Tanja Woyke, James Bristow, Jonathan A. Eisen, Victor Markowitz, Philip Hugenholtz, Nikos C. Kyrpides, Hans-Peter Klenk, Alla Lapidus

**Affiliations:** 1 DOE Joint Genome Institute, Walnut Creek, California, USA; 2 MTCC - Microbial Type Culture Collection, Institute of Microbial Technology, Chandigarh, India; 3 Los Alamos National Laboratory, Bioscience Division, Los Alamos, New Mexico, USA; 4 Biological Data Management and Technology Center, Lawrence Berkeley National Laboratory, Berkeley, California, USA; 5 Lawrence Livermore National Laboratory, Livermore, California, USA; 6 HZI – Helmholtz Centre for Infection Research, Braunschweig, Germany; 7 DSMZ - German Collection of Microorganisms and Cell Cultures GmbH, Braunschweig, Germany; 8 University of California Davis Genome Center, Davis, California, USA

**Keywords:** anaerobic, motile, vibrio-shaped, thiosulfate-reducing, H_2_S producing, peptide utilization, *Synergistaceae*, *Synergistetes*, GEBA

## Abstract

*Dethiosulfovibrio peptidovorans* Magot *et al.* 1997 is the type species of the genus *Dethiosulfovibrio* of the family *Synergistaceae* in the recently created phylum *Synergistetes*. The strictly anaerobic, vibriod, thiosulfate-reducing bacterium utilizes peptides and amino acids, but neither sugars nor fatty acids. It was isolated from an offshore oil well where it was been reported to be involved in pitting corrosion of mild steel. Initially, this bacterium was described as a distant relative of the genus *Thermoanaerobacter*, but was not assigned to a genus, it was subsequently placed into the novel phylum *Synergistetes*. A large number of repeats in the genome sequence prevented an economically justifiable closure of the last gaps. This is only the third published genome from a member of the phylum *Synergistetes*. The 2,576,359 bp long genome consists of three contigs with 2,458 protein-coding and 59 RNA genes and is part of the *** G****enomic* *** E****ncyclopedia of* *** B****acteria and* *** A****rchaea * project.

## Introduction

Strain SEBR 4207^T^ (= DSM 11002 = JCM 15826) is the type strain of the species *Dethiosulfovibrio peptidovorans* (‘curved rod-shaped [vibrio] bacterium that reduces thiosulfate devouring peptides’), which represents the type species of the genus *Dethiosulfovibrio* [1]. *D. peptidovorans* strain SEBR 4207^T^ was isolated in 1989 from an offshore oil well in the Congo (Brazzaville) and initially described by Magot *et al.* in 1997 [1]. The strain provided the first experimental evidence for the involvement of microbial thiosulfate reduction in the corrosion of steel (pitting corrosion). Strain SEBR 4207^T^ utilizes only peptides and amino acids, but no sugar or fatty acids. For the first few years neither the strain nor the genus *Dethiosulfovibrio* could be assigned to an established higher taxon, except that the distant relationship to the genus *Thermanaerovibrio* was reported [1]. The taxonomic situation of the species was only recently further enlightened, when Jumas-Bilak *et al*. [2] combined several genera with anaerobic, rod-shaped, amino acid degrading, Gram-negative bacteria into the novel phylum *Synergistetes* [2]. The phylum *Synergistetes* contains organisms isolated from humans, animals, terrestrial and oceanic habitats: *Thermanaerovibrio*, *Dethiosulfovibrio*, *Aminiphilus*, *Aminobacterium*, *Aminomonas*, *Anaerobaculum*, *Jonquetella*, *Synergistes* and *Thermovirga*. Given the novelty of the phylum it is not surprising that many of the type strains from these genera are already subject to genome sequencing projects. Here we present a summary classification and a set of features for *D. peptidovorans* strain SEBR 4207^T^, together with the description of the genomic sequencing and annotation.

## Classification and features

The 16S rRNA genes of the four other type strains in the genus *Dethiosulfovibrio* share between 94.2% (*D. salsuginis* [3]) and 99.2% (*D. marinus* [4]) sequence identity with strain SEBR 4207^T^, whereas the other type strains from the family *Synergistaceae* share 83.6 to 86.6% sequence identity [5]. There are no other cultivated strains that closely related. Uncultured clones with high sequence similarity to strain SEBR 4207^T^ were identified in a copper-polluted sediment in Chile (clones LC6 and LC23, FJ024724 and FJ024721, 99.1%). Metagenomic surveys and environmental samples based on 16S rRNA gene sequences provide no indication for organisms with sequence similarity values above 88% to *D. peptidovorans* SEBR 4207^T^, indicating that members of this species are not abundant in habitats screened thus far. The majority of these 16S rRNA gene sequences with similarity between 88% and 93% originate from marine metagenomes (status July 2010).

**Figure 1:**
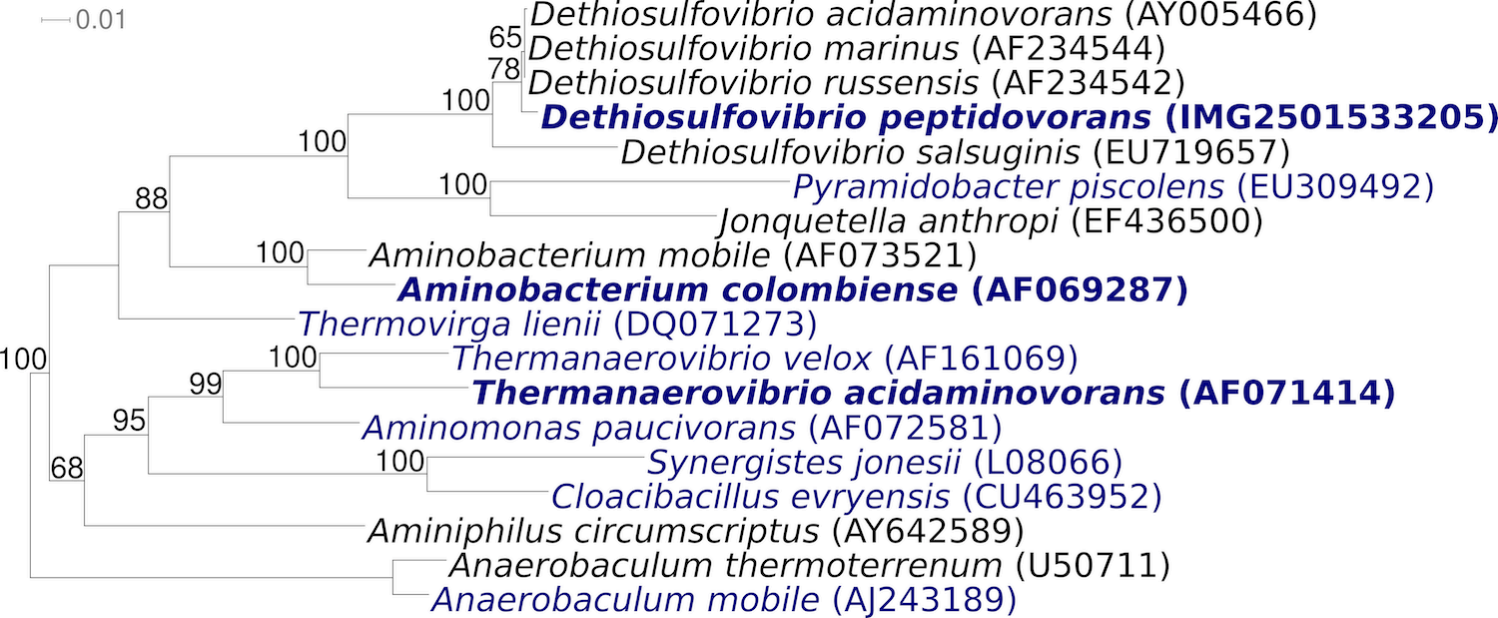
Phylogenetic tree highlighting the position of *D. peptidovorans* SEBR 4207^T^ relative to the other type strains within the phylum *Synergistetes*. The tree was inferred from 1,328 aligned characters [6,7] of the 16S rRNA gene sequence under the maximum likelihood criterion [8] and rooted in accordance with the current taxonomy [9]. The branches are scaled in terms of the expected number of substitutions per site. Numbers above branches are support values from 1,000 bootstrap replicates if greater than 60%. Lineages with type strain genome sequencing projects registered in GOLD [10] are shown in blue, published genomes in bold [11,12].

Figure 1 shows the phylogenetic neighborhood of *D. peptidovorans* SEBR 4207^T^ in a 16S rRNA based tree. The five copies of the 16S rRNA gene differ by up to one nucleotide from each other and by eight nucleotides from the previously published sequence generated from DSM 11002 (DPU52817).

**Figure 2:**
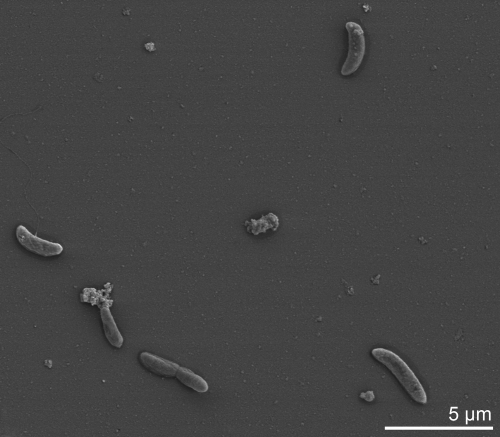
Scanning electron micrograph of *D. peptidovorans* SEBR 4207^T^

Cells of *D. peptidovorans* SEBR 4207^T^ stain Gram-negative [1]. Cells are vibriod with pointed or round ends and lateral flagella (Figure 2, flagella not visible) and a size of 3-5 by 1 µm [1] (Table 1). Spores were not detected [1]. Optimal growth rate was observed at 42°C, pH 7.0 in 3% NaCl [1]. *D. peptidovorans* is capable of utilizing peptides and amino acids as a sole carbon and energy source and can ferment serine and histidine. In the presence of thiosulfate, strain SEBR 4207^T^ is capable of utilizing alanine, arginine, asparagines, glutamate, isoleucine, leucine, methionine and valine as an electron acceptor. The strain is capable of producing acetate, isobutyrate, isovalerate, 2-methylbutyrate, CO_2_ and H_2_ from peptides. The strain uses elemental sulfur and thiosulfate but not sulfate as electron acceptor. H_2_S is produced with a decrease in H_2_. Cells do not have cytochrome or desulfoviridin [1]. When yeast extract was added as sole carbon and energy source together with trypticase, thiosulfate was used as sole electron acceptor. Strain SEBR 4207^T^ was not able to utilize gelatine, casein, arabinose, fructose, galactose, glucose, lactose, maltose, mannose, rhamnose, ribose, sucrose, sorbose, trehalose, xylose, acetate, propionate, butyrate, citrate and lactate.

**Table 1:** Classification and general features of *D. peptidovorans* SEBR 4207^T^ according to the MIGS recommendations [13].

**MIGS ID**	**Property**	**Term**	**Evidence code**
	Current classification	Domain *Bacteria*	TAS [14]
		Phylum *Synergistetes*	TAS [2]
		Class *Synergistia*	TAS [2]
		Order *Synergistales*	TAS [2]
		Family *Synergistaceae*	TAS [2]
		Genus *Dethiosulfovibrio*	TAS [1]
		Species *Dethiosulfovibrio peptidovorans*	TAS [1]
		Type strain SEBR 4207	TAS [1]
	Gram stain	negative	TAS [1]
	Cell shape	curved rods (vibrioid)	TAS [1]
	Motility	motile via lateral flagella	TAS [1]
	Sporulation	non-sporulating	TAS [1]
	Temperature range	mesophile, 20-45°C	TAS [1]
	Optimum temperature	42°C	TAS [1]
	Salinity	slightly halophilic, optimum 3% NaCl	TAS [1]
MIGS-22	Oxygen requirement	anaerobic	TAS [1]
	Carbon source	peptides and amino acids	TAS [1]
	Energy source	peptides and amino acids	TAS [1]
MIGS-6	Habitat	marine, oil wells	TAS [1]
MIGS-15	Biotic relationship	free living	NAS
MIGS-14	Pathogenicity	non pathogenic	NAS
	Biosafety level	1	TAS [15]
	Isolation	from corroding off-shore oil wells	TAS [1]
MIGS-4	Geographic location	Emeraude oil field, Congo (Brazzaville)	TAS [1]
MIGS-5	Sample collection time	before 1989	TAS [1]
MIGS-4.1 MIGS-4.2	Latitude Longitude	-5.05 11.78	NAS
MIGS-4.3	Depth	not reported	
MIGS-4.4	Altitude	about sea level	NAS

Evidence codes - IDA: Inferred from Direct Assay (first time in publication); TAS: Traceable Author Statement (i.e., a direct report exists in the literature); NAS: Non-traceable Author Statement (i.e., not directly observed for the living, isolated sample, but based on a generally accepted property for the species, or anecdotal evidence). These evidence codes are from of the Gene Ontology project [16]. If the evidence code is IDA, then the property was observed by one of the authors or an expert mentioned in the acknowledgements.

### Chemotaxonomy

None of the classical chemotaxonomic features (peptidoglycan structure, cell wall sugars, cellular fatty acid profile, menaquinones, or polar lipids) are known for *D. peptidovorans* SEBR 4207^T^ or any of the other members of the genus *Dethiosulfovibrio*.

## Genome sequencing and annotation

### Genome project history

This organism was selected for sequencing on the basis of its phylogenetic position [17], and is part of the *** G****enomic* *** E****ncyclopedia of* *** B****acteria and* *** A****rchaea * project [18]. The genome project is deposited in the Genome OnLine Database [10] and the complete genome sequence is deposited in GenBank. Sequencing, finishing and annotation were performed by the DOE Joint Genome Institute (JGI). A summary of the project information is shown in Table 2.

**Table 2:** Genome sequencing project information

**MIGS ID**	**Property**	**Term**
MIGS-31	Finishing quality	Permanent draft
MIGS-28	Libraries used	One 8 kb pMCL200 Sanger library, one 454 pyrosequence standard library and one Solexa library
MIGS-29	Sequencing platforms	ABI3730, 454 Titanium, Illumina GAii
MIGS-31.2	Sequencing coverage	8.0 x Sanger; 55.0 x pyrosequence
MIGS-30	Assemblers	Newbler version 1.1.02.15, Arachne
MIGS-32	Gene calling method	Prodigal 1.4, GenePRIMP
	INSDC ID	ABTR00000000
	Genbank Date of Release	May 1, 2009
	GOLD ID	Gc01332
	NCBI project ID	20741
	Database: IMG-GEBA	2501533205
MIGS-13	Source material identifier	DSM 11002
	Project relevance	Tree of Life, GEBA

### Growth conditions and DNA isolation

*D. peptidovorans* SEBR 4207^T^, DSM 11002, was grown anaerobically in DSMZ medium 786 (*Dethiosulfovibrio peptidovorans* Medium) [19] at 42°C. DNA was isolated from 0.5-1 g of cell paste using Qiagen Genomic 500 DNA Kit (Qiagen, Hilden, Germany) following the protocol as recommended by the manufacturer, with modification st/FT for cell lysis as described in Wu *et al.* [18].

### Genome sequencing and assembly

The genome was sequenced using a combination of Sanger and 454 sequencing platforms. All general aspects of library construction and sequencing can be found at the JGI website (http://www.jgi.doe.gov/). Pyrosequencing reads were assembled using the Newbler assembler version 1.1.02.15 (Roche). Large Newbler contigs were broken into overlapping fragments of 1,000 bp and entered into assembly as pseudo-reads. The sequences were assigned quality scores based on Newbler consensus q-scores with modifications to account for overlap redundancy and adjust inflated q-scores. A hybrid 454/Sanger assembly was made using Arachne assembler. Possible mis-assemblies were corrected and gaps between contgis were closed by primer walks off Sanger clones and bridging PCR fragments and by editing in Consed. A total of 392 Sanger finishing reads were produced to close gaps, to resolve repetitive regions, and to raise the quality of the finished sequence. Illumina reads were used to improve the final consensus quality using an in-house developed tool (the Polisher [20] ). The error rate of the final genome sequence is less than 1 in 100,000. Together, the combination of the Sanger and 454 sequencing platforms provided 63.0× coverage of the genome. The final assembly contains 35,314 Sanger reads and 626,193 pyrosequencing reads.

### Genome annotation

Genes were identified using Prodigal [21] as part of the Oak Ridge National Laboratory genome annotation pipeline, followed by a round of manual curation using the JGI GenePRIMP pipeline [22]. The predicted CDSs were translated and used to search the National Center for Biotechnology Information (NCBI) nonredundant database, UniProt, TIGRFam, Pfam, PRIAM, KEGG, COG, and InterPro databases. Additional gene prediction analysis and functional annotation was performed within the Integrated Microbial Genomes - Expert Review (IMG-ER) platform [23].

## Genome properties

**Figure 3:**
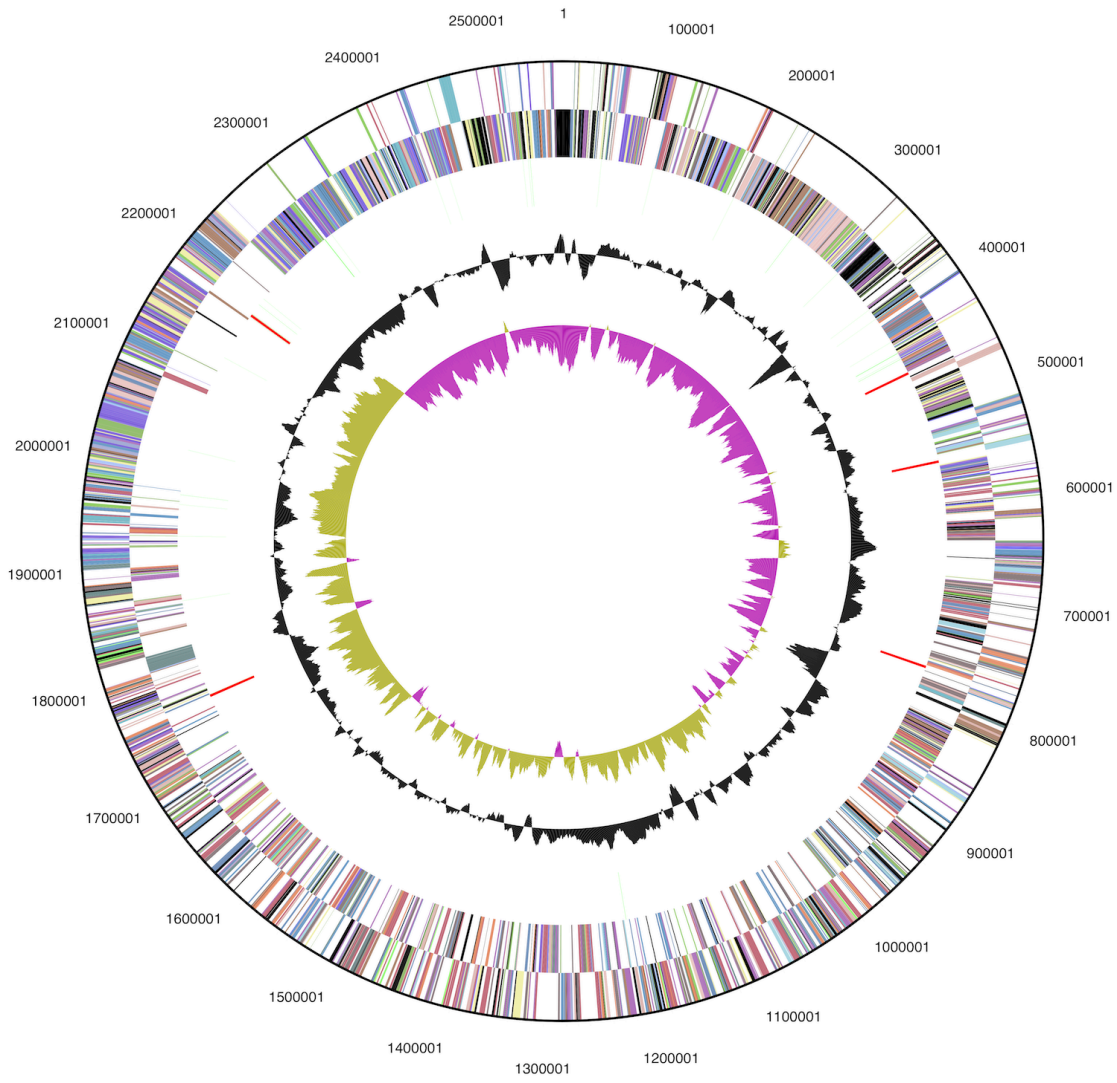
Graphical circular map of the genome (without the two small 1.5 and 7.4 kbp plasmids. From outside to the center: Genes on forward strand (color by COG categories), Genes on reverse strand (color by COG categories), RNA genes (tRNAs green, rRNAs red, other RNAs black), GC content, GC skew.

The genome is 2,576,359 bp long and assembled in one large contig and two small contigs (7,415 bp and 1,508 bp) with a 54.0% G+C content (Table 3 and Figure 3). Of the 2,517 genes predicted, 2,458 were protein-coding genes, and 59 RNAs; No pseudogenes were identified. The majority of the protein-coding genes (75.0%) were assigned with a putative function while the remaining ones were annotated as hypothetical proteins. The distribution of genes into COGs functional categories is presented in Table 4.

**Table 3:** Genome Statistics

**Attribute**	**Value**	**% of Total**
Genome size (bp)	2,576,359	100.00%
DNA coding region (bp)	2,391,158	92.81%
DNA G+C content (bp)	1,401,945	54.42%
Number of repolicons	3	
Extrachromosomal elements	2	
Total genes	2,517	100.00%
RNA genes	59	1.40%
rRNA operons	5	
Protein-coding genes	2,458	97.27%
Pseudo genes	0	0.00%
Genes with function prediction	1,888	75.01%
Genes in paralog clusters	438	17.41%
Genes assigned to COGs	1,952	77.55%
Genes assigned Pfam domains	2,007	79.74%
Genes with signal peptides	420	16.69%
Genes with transmembrane helices	619	24.59%
CRISPR repeats	2	

**Table 4:** Number of genes associated with the general COG functional categories

**Code**	**value**	**%age**	**Description**
J	149	6.7	Translation, ribosomal structure and biogenesis
A	0	0.0	RNA processing and modification
K	129	5.9	Transcription
L	115	5.3	Replication, recombination and repair
B	0	0.0	Chromatin structure and dynamics
D	28	1.3	Cell cycle control, mitosis and meiosis
Y	0	0.0	Nuclear structure
V	32	1.5	Defense mechanisms
T	133	6.1	Signal transduction mechanisms
M	119	5.5	Cell wall/membrane biogenesis
N	75	3.5	Cell motility
Z	0	0.0	Cytoskeleton
W	0	0.0	Extracellular structures
U	46	2.1	Intracellular trafficking and secretion, and vesicular transport
O	70	3.2	Posttranslational modification, protein turnover, chaperones
C	142	6.5	Energy production and conversion
G	113	5.2	Carbohydrate transport and metabolism
E	252	11.6	Amino acid transport and metabolism
F	65	3.0	Nucleotide transport and metabolism
H	99	4.6	Coenzyme transport and metabolism
I	44	2.0	Lipid transport and metabolism
P	125	5.8	Inorganic ion transport and metabolism
Q	31	1.4	Secondary metabolites biosynthesis, transport and catabolism
R	243	11.2	General function prediction only
S	161	7.4	Function unknown
-	565	22.5	Not in COGs
